# Huang-Pu-Tong-Qiao Formula Ameliorates Tau Phosphorylation by Inhibiting the CaM-CaMKIV Pathway

**DOI:** 10.1155/2020/8956071

**Published:** 2020-05-11

**Authors:** Shu Ye, Biao Cai, Peng Zhou, Guoquan Wang, Huawu Gao, Rupeng Hua, Liangzhen You, Yongchuan Yao, Yan Wang, Guoming Shen

**Affiliations:** ^1^School of Integrated Chinese and Western Medicine, Anhui University of Chinese Medicine, Hefei 230012, Anhui, China; ^2^Institute of Integrated Chinese and Western Medicine, Anhui Academy of Chinese Medicine, Hefei 230012, Anhui, China; ^3^Anhui Province Key Laboratory of Chinese Medicinal Formula, Hefei 230012, Anhui, China; ^4^The First Affiliated Hospital of Anhui University of Traditional Chinese Medicine, Hefei 230031, Anhui, China

## Abstract

Alzheimer's disease (AD) is a complex neurodegenerative disease. It is a chronic, lethal disease in which brain function is severely impaired and neuronal damage is irreversible. Huang-Pu-Tong-Qiao (HPTQ), a formula from traditional Chinese medicine, has been used in the clinical treatment of AD for many years, with remarkable effects. However, the neuroprotective mechanisms of HPTQ in AD have not yet been investigated. In the present study, we used AD models *in vivo* and *in vitro*, to investigate both the neuroprotective effect of HPTQ water extracts (HPTQ-W) and the potential mechanisms of this action. For the *in vivo* study, after HPTQ intervention, the Morris water maze test was used to examine learning and memory in rats. Transmission electron microscopy and immunofluorescence methods were then used to investigate neuronal damage. For the *in vitro* experiments, rat primary hippocampal neurons were cultured and cell viability was examined by 3-(4,5-dimethylthiazol-2-yl)-2,5-diphenyltetrazolium bromide. Additionally, mRNA levels of CaM, CaMKK, CaMKIV, and tau were examined using qRT-PCR, and protein expression of CaM, CaMKK, p-CaMKIV, and p-tau were examined using western blot. *In vivo*, we revealed that HPTQ significantly improved learning and memory deficits and attenuated neuronal damage in the AD rat model. Furthermore, *in vitro* results showed that HPTQ significantly increased cell viability in the AD cell model. We also demonstrated that HPTQ significantly decreased the mRNA levels of CaM, CaMKK, CaMKIV, and tau and significantly decreased the protein expressions of CaM, CaMKK, p-CaMKIV, and p-tau. In conclusion, our results indicated that HPTQ improved cognition and ameliorated neuronal damage in AD models and implicated a reduction in tau phosphorylation caused by inhibition of the CaM-CaMKIV pathway as a possible mechanism.

## 1. Introduction

Alzheimer's disease (AD) is an age-related neurodegenerative disease [1] that is characterized by cognitive deficits, memory loss, language impairments, and mental and behavioral alterations [2, 3]. Extracellular A*β* deposition forms senile plaques, and intracellular hyperphosphorylated tau produces neurofibrillary tangles (NFTs); these are the two hallmark histopathological lesions of AD. The World Alzheimer's Report of 2018 reported that 50 million people worldwide were living with dementia in 2018, and this number is expected to more than triple to 152 million by 2050. The cost of dementia is enormous, having reached $1 trillion by 2018, and is still increasing [4]. However, the mechanisms of AD are complex and may include the cholinergic hypothesis, the amyloid-beta (A*β*) hypothesis, the tau hypothesis, the neurotoxicity hypothesis, and the oxidative stress hypothesis, among others [5].

Tau is a microtubule-associated protein that plays an important role in the synthesis and stabilization of the neuronal cytoskeleton to maintain cell integrity [6]. Abnormal hyperphosphorylation of tau (p-tau) decreases the binding affinity of microtubules, resulting in NFTs. These changes have been associated with multiple neurodegenerative disorders and are closely related to AD severity [7, 8]. Tau can be phosphorylated in 85 residues [9], by kinases such as glycogen synthase kinase 3 (GSK-3), mitogen-activated protein kinase (MAPK), and Ca^2+^-calmodulin-dependent protein kinase II (CaMKII) [10]. These kinases are considered to be targets for anti-p-tau therapies. Studies have shown that Tideglusib, a GSK-3 inhibitor, improves cognition in double transgenic mice expressing human tau and amyloid precursor protein (APP) by reducing tau phosphorylation, amyloid deposition, and neuronal loss [11].

However, the treatment of AD has historically been very difficult and remains challenging in the present. There are currently four FDA-approved medications for improving AD symptoms, including cholinesterase inhibitors, such as donepezil, and the uncompetitive NMDA receptor modulator memantine. In addition, some novel approaches (including those involving BChE inhibition, antiamyloid agents, tau protein and related enzymes, and antioxidants) can alleviate patients' conditions, but do not modify AD progression. So far, amyloid-based therapies have fallen into the bottleneck in improving the course of AD, and tau-oriented therapies are still in the early stages of development [12].

Huang-Pu-Tong-Qiao (HPTQ) is a hospital formula from the First Affiliated Hospital of Anhui University of Traditional Chinese Medicine and is mainly composed of Shi Chang-pu, Yi-zhi, He Shou-wu, Chuang-xiong, Da-huang, and Ren-sheng. This formula has been used to treat dementia for more than 10 years and has a remarkable curative effect; however, the mechanism of this effect is unclear. We hypothesized that HPTQ might reduce tau hyperphosphorylation, thus achieving its effective treatment of AD. To elucidate the mechanism of HPTQ in the treatment of AD, we used intraperitoneal injections of D-galactose combined with intraventricular injections of A*β*_25–35_ to establish an AD animal model and used A*β*_25–35_ to induce an AD cell model in primary hippocampal neurons. Histopathological changes and cellular biomarkers were examined.

## 2. Materials and Methods

### 2.1. Materials

Stilbene glycoside, ferulic acid, ginsenoside Rg1, aloe-emodin, *β*-asarone, emodin, and chrysophanol were purchased from China National Institutes for Food and Drug Control (Beijing, China). Nimodipine was purchased from Bayer Medical and Health Co., Ltd. (Beijing, China). Neurobasal-A media (NM) and B27 were purchased from Gibco Industries Inc. (Auckland, New Zealand). Poly-D-lysine (PDL) and A*β*_25-35_ were purchased from Sigma-Aldrich (St. Louis, MO, USA). KN-62 was purchased from Selleck Chemicals (Houston, TX, USA).

### 2.2. A*β* Aggregation

A*β*_25–35_ was added to sterile double-distilled water at a concentration of 2 mg/mL or 1 mg/mL as the mother liquor for the *in vivo* and *in vitro* assays, respectively. The mother liquor was incubated at 37°C for 4 days and then stored at −20°C.

### 2.3. Preparation and Ultra-Performance Liquid Chromatography (UPLC) Analysis of HPTQ

The HPTQ solution was prepared fresh every 3 days by boiling the following six components in water: Shi Chang-pu (20 g), Yi-zhi (20 g), He Shou-wu (20 g), Chuang-xiong (20 g), Da-huang (6 g), and Ren-sheng (10 g). Tenfold water was added and refluxed for 2 h for the first time, and for the second time, 8-fold water was added and refluxed for 1.5 h. The solution was then collected, concentrated to 50 mg/mL, and stored at 4°C.

Stilbene glycoside, ferulic acid, ginsenoside Rg1, aloe-emodin, *β*-asarone, emodin, and chrysophanol levels in HPTQ were measured using UPLC. The Acquity BEH C18 (2.1 mm × 100 mm, 1.7 *μ*m; Waters, Milford, MA, USA) analytical column was coupled with a column filter, and the temperature was set at 30°C. The gradient elution with two mobile phase systems consisting of 0.05% (v/v) phosphoric acid in distilled water (A) and acetonitrile (B) was performed as follows: 5%–25% B for 0–2 min, 25%–67% B for 2–8 min, 67%–82% B for 8–10 min, and 82%–5% B for 10–12 min, with a reequilibrium time of 2 min. The flow rate and injection volume were 0.2 mL/min and 1 *μ*L, respectively. The (photodiode array, PDA) detector wavelength was monitored at 210 nm.

### 2.4. Neuroprotective Effect of HPTQ on AD *In Vivo*

#### 2.4.1. Animals and Treatments

A total of 40 male Sprague Dawley rats (250–300 g, 8–10 months old) were sourced from the Animal Experiment Center, Anhui Medical University, China (Certificate no. SXCK (Wan) 2011–002). The rats were fed adaptively for a week under a light/dark cycle of 12 h/12 h and kept at a temperature of 25°C–30°C.

After excluding animals with outlying results in the Morris water maze test, the rats were randomly divided into four groups: the normal control group (CTRL), the model group (MODEL), the HPTQ group (1.41 g/kg, corresponding to three times the clinical equivalent according to the volume surface area folding algorithm), and the nimodipine group (NMDP; 20 mg/kg, corresponding to three times the clinical equivalent according to the volume surface area folding algorithm). Except for the CTRL group, rats in the other groups were intraperitoneally injected with 100 mg/kg D-galactose once daily for 42 days. When they had undergone 21 days of D-galactose treatment, the rats were fastened to a brain stereotaxic instrument (Stoelting Co., Wood Dale, IL, USA) and injected using a microsyringe (Anting Scientific Instrument, Shanghai, China) with 5 *μ*L (10 *μ*g) A*β*_25–35_ into the bilateral hippocampus. The injection point was located at 4.4 mm posterior to and 2.2 mm from the anterior fontanelle as the origin point, and the needle was placed 3.0 mm from the rat hippocampus [13]. The CTRL group was injected with an equal volume of saline. After treatment with D-galactose for 14 days, the HPTQ group and NMDP group rats were fed by gavage according to body mass.

#### 2.4.2. Morris Water Maze Test

After intracerebral injection of A*β*_25–35_ for 7 days in rats, the classical Morris water maze method was used [14]. The experiment lasted for 6 days. For the first 5 days, the navigation experiment was performed. Rats were placed into the water from one of four quadrants, facing the wall of the pool, and the time taken to find the platform within 90 s was recorded. If the rats did not find the platform within 90 s, they were placed on the platform and left there for 20 s. On day 5, no guidance was given, and the time taken to find the platform was recorded as the escape latency. On day 6, the platform was removed and the same parameters were set as for the navigation experiments. The number of times that the rats crossed the area where the platform had been located was recorded.

#### 2.4.3. Animal Sample Preparation

After the Morris water maze test, rats were sacrificed by anesthesia with an intraperitoneal injection of 1% sodium pentobarbital (40 mg/kg). Brain tissue was quickly removed and placed on saline ice. Some hippocampal tissue was cut to 1 mm^3^ size and stored in glutaraldehyde, to be used in transmission electron microscopy (TEM) experiments, and the remaining hippocampal tissue was isolated from both sides of the brain and stored at −80°C for western blot and qRT-PCR experiments.

#### 2.4.4. Determination of the Fluorescent Expression of p-Tau

Brain tissue was fixed in 4% paraformaldehyde, then dehydrated, embedded, sectioned, and placed into pH 8.8 for repair and sealing. Tissue was then incubated overnight at 4°C in primary rabbit anti-rat p-tau antibody. The following day, the brain tissue was incubated in fluorescent secondary FITC-rabbit anti-goat IgG antibody at room temperature for 2 h, washed three times in phosphate-buffered solution (PBS), and incubated in DAPI for 10 min. After washing the slides, an antifluorescent quencher was used to coverslip the tissue, and images were collected using an inverted fluorescence microscope.

### 2.5. Effect of HPTQ on AD *In Vitro*

#### 2.5.1. Cell Culture and Intervention

The methods of culturing primary hippocampal neurons were performed according to Korkotian et al. [15, 16]. Neonatal pups were sacrificed after immersion in 75% alcohol for 20 s. Hippocampi were dissected out and cut into 1 mm^3^ pieces after separating the blood vessels and meninges. The tissue was digested with 0.125% trypsin for 20 min at 37°C, before DMEM/F12 medium with 10% fetal bovine serum (FBS) was added to terminate the digestion. The cells were separated and collected by centrifuging the cell suspension at 1500 rpm for 5 min. Next, the suspension was swirled gently and filtered through a 200-mesh sieve. Isolated primary hippocampal neurons were cultured in DMEM/F12 nutrient solution containing 20% FBS. Subsequently, the cells were seeded onto poly-D-lysine-coated plates at a density of 1 × 10^6^/mL and maintained at 37°C in an incubator with 5% CO_2_. The medium was replaced with NM supplemented with 2% B27 and 0.5 mM/L L-glutamine, and half of the medium was changed every 3 days.

**Figure 1:**
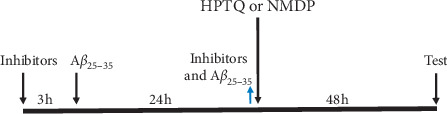
The time axis of drug intervention in hippocampal neurons. 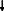: added; 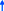: removed.

Based on previous experimental results from our research group, the neurons were assigned to the following treatment groups after being cultured for 7 days: the control group (CTRL), model group (MODEL), KN-62 group (KN62), 15% HPTQ-medicated serum group (HPTQ), 15% HPTQ-medicated serum + KN-62 group (KN62 + HPTQ), and nimodipine group (NMDP). Cells in the MODEL group were incubated with 30 *μ*M A*β*_25–35_ for 24 h to establish the AD cell model. Cells in the KN-62 group were incubated with 10 *μ*M of the KN-62 inhibitor for 3 h before the AD cell model was established. Cells in the HPTQ and NMDP groups were given 15% HPTQ-medicated serum and 7 *μ*M nimodipine solution, respectively, for 24 h after the establishment of the AD cell model. Cells in the KN62 + HPTQ group were given 15% HPTQ-medicated serum for 24 h after being preadministrated 10 *μ*M KN-62 and the establishment of the AD cell model. Groups are shown in Figure 1.

#### 2.5.2. Cell Viability

After the cells were treated and cultured at 37°C in a 5% CO_2_ incubator, the supernatant was removed and 20 *μ*L of 5 mg/mL MTT was added to each well and incubated for 4 h. The supernatant was removed and 150 *μ*L DMSO was added to each well. The optical density at 490 nm was measured using a microplate reader (318C+, Shanghai Peiou Co., Ltd., Shanghai, China).

### 2.6. Mechanism of HPTQ on AD *In Vitro* and *In Vivo*

#### 2.6.1. Quantitative Real-Time PCR (qRT-PCR)

The total RNA of hippocampal tissue or treated cells was extracted according to the instructions using a TRIzol kit (Invitrogen, Carlsbad, CA, USA). cDNA was synthesized from total cellular RNA using a RevertAid First Strand cDNA Synthesis Kit (Thermo Fisher Scientific, San Jose, CA, USA). qRT-PCR was performed using an ABI PRISM 7500 System (Applied Biosystems, Foster City, CA, USA; Life Technologies) and QuantiNova SYBR Green PCR Kit (Qiagen, Dusseldorf, Germany). The mRNA levels of CaM, CaMKK, CaMKIV, tau, and *β*-actin in hippocampal tissues and neurons were measured using qRT-PCR. The expression levels of each gene were calculated using the 2^−ΔΔCT^ method [17]. The mRNA expression levels were displayed as fold changes compared with the internal control, *β*-actin. The primer sequences are shown in Table 1.

#### 2.6.2. Western Blot Analysis

The total cellular protein was extracted from hippocampal tissue or treated cells using a protein extraction kit, and the concentrations were measured using a Bicinchoninic Acid Kit (Beyotime Biotechnology, Shanghai, China). Equal amounts of protein were separated by 10%–12% SDS-PAGE (Bio-Rad) and transferred onto polyvinylidene fluoride (PDVF) membranes (Millipore, Billerica, MA, USA). After subsequently blocking in 5% nonfat dry milk for 2 h, the membranes were incubated overnight at 4°C with the relevant primary antibody, followed by incubation with secondary antibodies for 1.5 h at 37°C. The primary rabbit anti-rat monoclonal antibodies against CaM, CaMKK, p-CaMKIV, and p-tau were all obtained from Abcam (1 : 1000, Cambridge, UK). The secondary antibody, HRP-conjugated goat anti-rabbit IgG, was obtained from AmyJet Scientific Inc. (1 : 20,000, Abbkine, Wuhan, China). Rabbit anti-rat *β*-actin (1 : 2000, Abbkine, AmyJet Scientific Inc.) was used as an internal control. The protein bands were visualized using electrogenerated chemiluminescence (ECL; Thermo Fisher Scientific) and analyzed on a gel imager (FluorChem M, ProteinSimple, USA).

### 2.7. Statistical Analysis

The measured data are presented as mean ± standard deviation (SD). Multiple group means were compared by one-way analysis of variance and the LSD method. All analyses were performed using SPSS version 23.0 software, with a significance level set at *P* < 0.05.

## 3. Results

### 3.1. Qualitative Analysis of HPTQ

**Figure 2:**
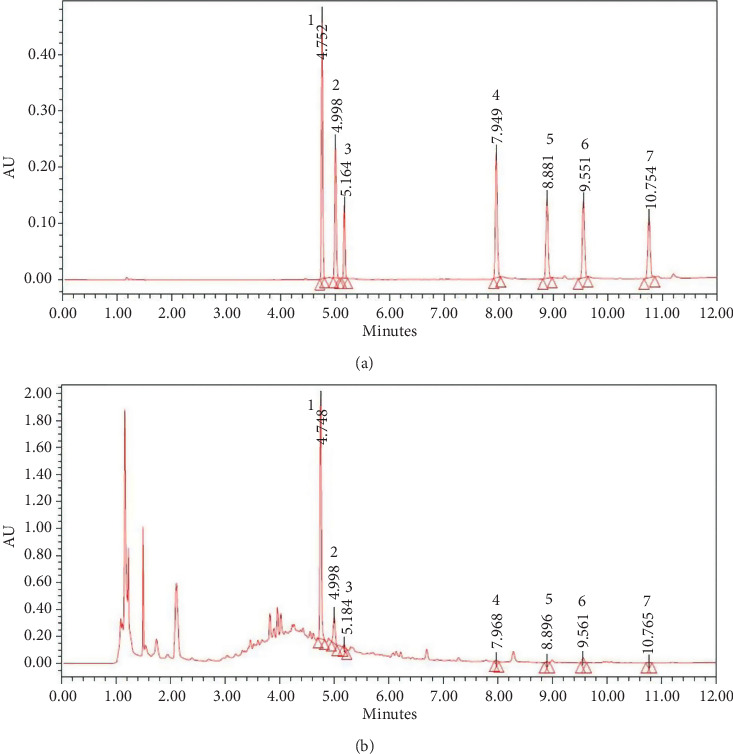
Peak chromatograms of Huang-Pu-Tong-Qiao (HPTQ). Mixed standard chromatogram: (1) stilbene glycoside; (2) ferulic acid; (3) ginsenoside Rg1; (4) aloe-emodin; (5) *β*-asarone; (6) emodin; (7) chrysophanol (a) and HPTQ chromatogram (b).

Stilbene glycoside, ferulic acid, ginsenoside Rg1, aloe-emodin, *β*-asarone, emodin, and chrysophanol in HPTQ were determined by UPLC (Figure 2).

### 3.2. Protective Effect of HPTQ on Neuronal Damage and Memory Improvement in AD Model Rats

#### 3.2.1. Learning and Memory in AD Model Rats

**Figure 3:**
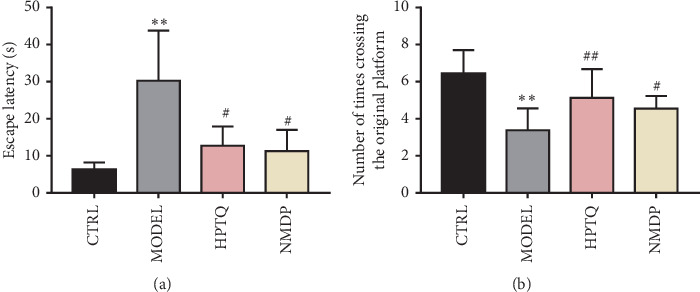
Effect of HPTQ on learning and memory in AD model rats. Effect of HPTQ on escape latencies in AD model rats (a). The number of times of crossing the original platform location in AD model rats (b). Values are expressed as the mean ± SD. ^*∗*^*P* < 0.05, ^*∗∗*^*P* < 0.01, vs. CTRL group; ^#^*P* < 0.05, ^##^*P* < 0.01, vs. MODEL group.

The Morris water maze was performed to evaluate spatial reference memory. In the escape latency test, the time taken to reach the platform was significantly higher in the MODEL group rats than in the CTRL group rats (*P* < 0.01). However, compared with the MODEL group rats, the time taken to reach the platform was significantly lower in the HPTQ and NMDP group rats (*P* < 0.05 and *P* < 0.01). When comparing the number of times crossing the original platform, MODEL group rats had significantly lower frequencies than the CTRL group rats (*P* < 0.05 or *P* < 0.01). Furthermore, compared with the MODEL group rats, the frequencies were significantly higher in the HPTQ and NMDP group rats (Figure 3).

#### 3.2.2. Effect of HPTQ on Hippocampal CA3 Region Ultrastructure in AD Rats

**Figure 4:**
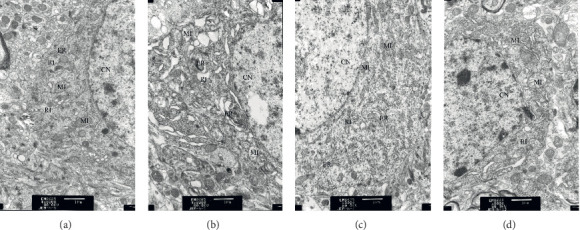
Effect of HPTQ against A*β*25-35-induced neuronal impairment in AD rats. CTRL group (a). MODEL group (b). HPTQ group (c). NMDP group (d). CN: nucleus; RI: ribosome; MI: mitochondria; ER: endoplasmic reticulum.

The TEM results revealed that, in the CTRL group, tissue characteristics included intact nuclear membranes and condensed chromatin, with abundant organelles in the cytoplasm; in particular, a large number of ribosomes and rough endoplasmic reticulum were clearly visible. The cristae of mitochondria were intact, normal in structure, and without damage. In the MODEL group, the structure of neuronal nuclear membranes was blurred and irregular, and there were cytoplasmic edema and severe organelle damage. The mitochondria were swollen with some broken cristae, and vacuolar degeneration was apparent. In addition, most rough endoplasmic reticula were dilated, empty gun state, and even partly disappeared, with greatly reduced numbers of ribosomes. In the HPTQ and NMDP groups, nuclear membranes were relatively intact, and there were more ribosomes in the rough endoplasmic reticulum in the cytoplasm of neurons. A small number of mitochondria were slightly swollen and their cristae processes were destroyed (Figure 4).

### 3.3. Effect of HPTQ on Fluorescent p-Tau Expression in AD Rats

**Figure 5:**
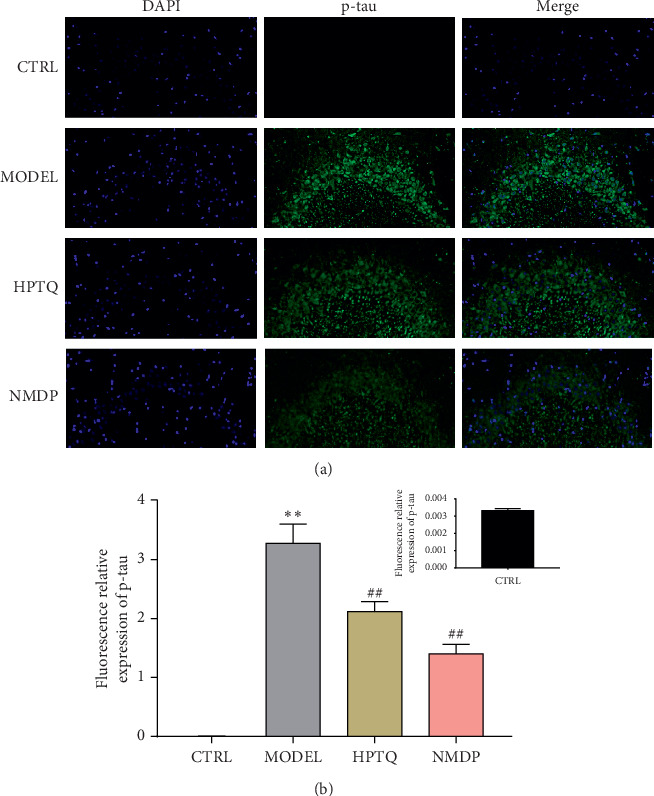
Effect of HPTQ on fluorescent protein expression of p-tau in AD rats. Immunofluorescence microscopic images of the CA3 region of the hippocampus (a). p-Tau^+^ area/DAPI^+^ area (b). Values are expressed as the mean ± SD. ^*∗*^*P* < 0.05, ^*∗∗*^*P* < 0.01, vs. CTRL group; ^#^*P* < 0.05, ^##^*P* < 0.01, vs. MODEL group.

Immunofluorescence was used to detect the fluorescent protein expression of p-tau in the hippocampal CA3 region of AD rats. Compared with the CTRL group, the fluorescent expression of p-tau was significantly higher in the MODEL group, although the number of neurons was lower. However, compared with the MODEL group, the fluorescent expression of p-tau in the HPTQ and NMDP groups was significantly lower, while the number of neurons was higher (Figure 5).

### 3.4. Protective Effect of HPTQ-Medicated Serum on A*β*_25–35_-Induced Neurotoxicity in Rat Hippocampal Neurons

**Figure 6:**
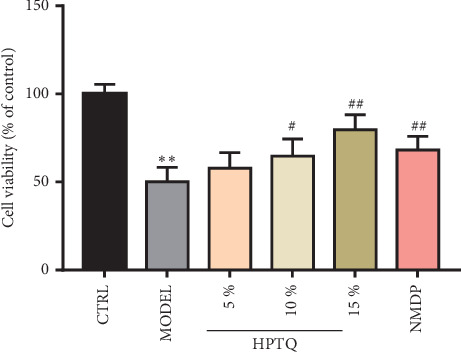
Protective effect of Huang-Pu-Tong-Qiao (HPTQ)-medicated serum on the cell survival rate induced by A*β*_25–35._ Values are expressed as the mean ± SD. ^*∗*^*P* < 0.05, ^*∗∗*^*P* < 0.01, vs. CTRL group; ^#^*P* < 0.05, ^##^*P* < 0.01, vs. MODEL group.

To further determine the protective effect of HPTQ-medicated serum in rat hippocampal neurons, cell viability and cytotoxicity were measured using MTT. After the establishment of a cell model induced by A*β*_25–35_, either 5%, 10%, or 15% HPTQ-medicated serum and nimodipine solution was continued to intervene for another 24 h. Compared with the CTRL group, the cell survival rate was significantly lower in the MODEL group (*P* < 0.01). However, compared with the MODEL group, the cell survival rate was significantly higher in the 10% and 15% HPTQ-medicated serum groups and the NMDP group (*P* < 0.05 or *P* < 0.01; Figure 6).

### 3.5. Effect of HPTQ on Relevant mRNA Levels in the CaM-CaMKIV Pathway in AD *In Vivo* and *In Vitro* Models

**Figure 7:**
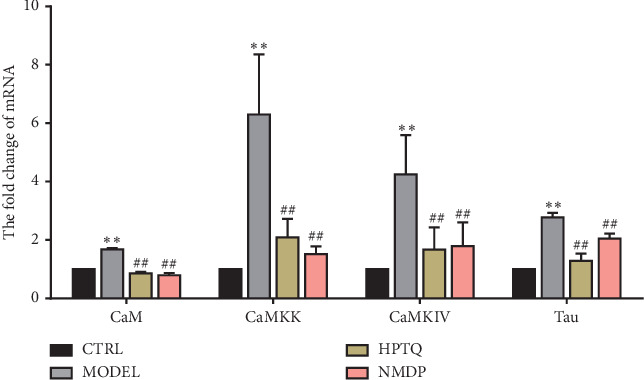
Effect of Huang-Pu-Tong-Qiao (HPTQ) on mRNA levels of CaM, CaMKK, CaMKIV, and tau in AD model rats. Values are expressed as the mean ± SD (*n*=3). ^*∗*^*P* < 0.05, ^*∗∗*^*P* < 0.01, vs. CTRL group; ^#^*P* < 0.05, ^##^*P* < 0.01, vs. MODEL group.

**Figure 8:**
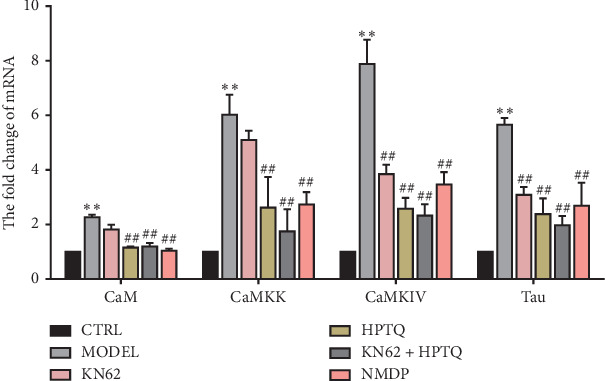
Effect of Huang-Pu-Tong-Qiao- (HPTQ-) medicated serum on mRNA levels of CaM, CaMKK, CaMKIV, and tau in primary hippocampal neurons. Values are expressed as the mean ± SD (*n*=3). ^*∗*^*P* < 0.05, ^*∗∗*^*P* < 0.01, vs. CTRL group; ^#^*P* < 0.05, ^##^*P* < 0.01, vs. MODEL group.

The mRNA expressions of CaM, CaMKK, CaMKIV, and tau were significantly higher in the MODEL group tissue compared with the CTRL group (*P* < 0.01). Compared with the MODEL group, the mRNA expressions of CaM, CaMKK, CaMKIV, and tau in the HPTQ and NMDP groups were significantly lower (*P* < 0.01; Figure 7). To further identify the role of the CaM-CaMKIV pathway in tau hyperphosphorylation, we used KN-62, a CaMKIV inhibitor [18], either separately or as a cotreatment with HPTQ in a cell culture model. Compared with the CTRL group, the mRNA levels of CaM, CaMKK, CaMKIV, and tau were significantly higher in the MODEL group (*P* < 0.01). Compared with the MODEL group, the mRNA expressions of CaM and CaMKK in the KN62 group were not statistically different, while the expression levels of CaMKIV and tau mRNA were significantly lower (*P* < 0.01). Furthermore, compared with the MODEL group, the mRNA levels of CaM, CaMKK, CaMKIV, and tau in the HPTQ, KN62 + HPTQ, and NMDP groups were significantly lower (*P* < 0.01; Figure 8).

### 3.6. Effect of HPTQ on Relevant Protein Expression Levels in the CaM-CaMKIV Pathway in AD *In Vivo* and *In Vitro* Models

**Figure 9:**
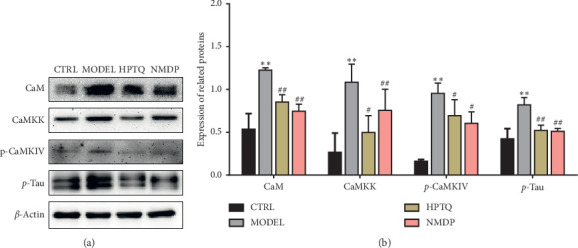
Effect of HPTQ on the protein expressions of CaM, CaMKK, p-CaMKIV, and p-tau in the *in vivo* AD model. The gray bands of different proteins (a). The graph displays densitometric analyses of the expression ratios of CaM, CaMKK, p-CaMKIV, and p-tau (b). Values are expressed as the mean ± SD (*n*=3). ^*∗*^*P* < 0.05, ^*∗∗*^*P* < 0.01, vs. CTRL group; ^#^*P* < 0.05, ^##^*P* < 0.01, vs. MODEL group.

**Figure 10:**
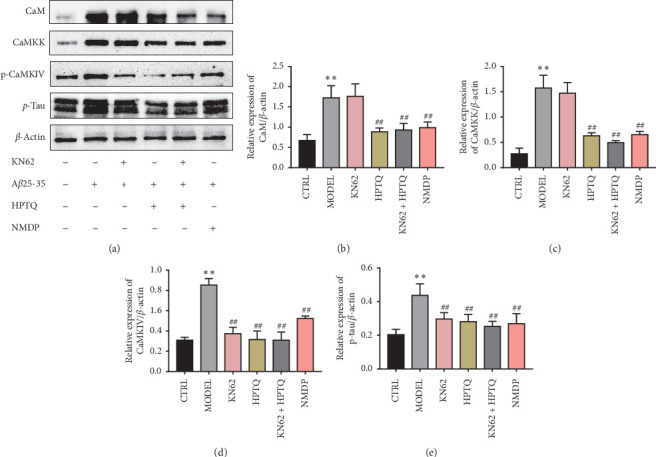
Effect of HPTQ on the protein expressions of CaM, CaMKK, p-CaMKIV, and p-tau in the *in vitro* AD model. The gray bands of different proteins (a). The graph displays densitometric analyses of the expression ratios of CaM, CaMKK, p-CaMKIV, and p-tau (b). Values are expressed as the mean ± SD (*n*=3). ^*∗*^*P* < 0.05, ^*∗∗*^*P* < 0.01, vs. CTRL group; ^#^*P* < 0.05, ^##^*P* < 0.01, vs. MODEL group.

In rat tissue, the protein expressions of CaM, CaMKK, p-CaMKIV, and p-tau were significantly higher in the MODEL group than in the CTRL group (*P* < 0.01). Compared with the MODEL group, the protein expressions of CaM, CaMKK, p-CaMKIV, and p-tau in HPTQ and NMDP group were significantly lower (*P* < 0.01; Figure 9). After separate or cotreatment with KN-62 in a cell culture model, compared with the CTRL group, the protein expressions of CaM, CaMKK, p-CaMKIV, and p-tau protein in the MODEL group were significantly higher (*P* < 0.01). Compared with the MODEL group, the protein expressions of CaM and CaMKK in the KN62 group were not statistically different, while the protein expressions of CaM and CaMKK in the HPTQ, KN62 + HPTQ, and NMDP groups were significantly lower (*P* < 0.01). Compared with the MODEL group, the protein expressions of p-CaMKIV and p-tau were significantly lower in the KN62, HPTQ, KN62 + HPTQ, and NMDP groups (*P* < 0.01; Figure 10).

## 4. Discussion

In the present study, we investigated the neuroprotective effect of HPTQ against A*β*-induced toxicity in rats and rat hippocampal neurons. We also examined the CaM-CaMKIV pathway mediation of tau phosphorylation. The results revealed that HPTQ improved hippocampal neuronal damage and increased cell viability. In addition, HPTQ decreased tau phosphorylation via inhibition of the CaM-CaMKIV pathway. In addition, the use of KN-62, a CaMKIV inhibitor, further demonstrated that HPTQ decreases tau phosphorylation via inhibition of the CaM-CaMKIV pathway.

Extracellular senile plaques, intracellular NFTs, and neuronal loss are the characteristic pathologies of AD, which lead to learning and memory impairments [19]. The main component of senile plaques is A*β*, forming insoluble fibrillar deposits in AD patients [20], and these mediate neuronal degeneration and loss. A previous study demonstrated that an A*β* injection into the rodent hippocampus results in physiological and cognitive deficits [21]. In addition, A*β* is extensively used to induce *in vivo* and *in vitro* models of AD because it is a neurotoxin [22]. A*β*_25–35_ forms *β*-sheet structures and amyloid-like fibrils and is likely endogenously produced by the enzymatic cleavage of A*β*_1–40_ when injected into the lateral ventricle [23]. In the present study, we used an intraperitoneal injection of 100 mg/kg D-galactose, combined with a lateral ventricle injection of 2 mg/mL A*β*_25–35_, to establish an AD rat model. We also used 30 *μ*M A*β*_25–35_ to induce the establishment of an AD cell model. We then demonstrated that HPTQ promoted learning and memory in AD rats and increased cell viability.

We also specifically examined the CaM-CaMKIV pathway-mediated reduction of tau phosphorylation. In AD, there is a wide imbalance in Ca^2+^ signaling homeostasis [24]. Previous research has shown that A*β* enhances Ca^2+^ entry and that its overload aggravates Ca^2+^ imbalance [25]. CaM is a cytosolic protein that is expressed in all eukaryotic cells. It is the main receptor for Ca^2+^ in cells and combines with Ca^2+^ to form a Ca^2+^/CaM complex that participates in modulating cellular signaling pathways [26]. CaMK is one kind of signaling protein that is regulated by Ca^2+^/CaM. Multifunctional CaMKs are abundant in the brain and are activated by combining with Ca^2+^/CaM, such as members of the CaMK cascade (CaMKK and CaMKIV) that alter and regulate the functionality of numerous protein substrates by phosphorylating them. For example, CaMKIV can be activated by CaMKK phosphorylating the kinase in its activation loop on Thr196 [27]. CaMKIV has a two-way regulatory function and can either promote or inhibit cell proliferation by regulating transcription factors. The multifunctional CaMKIV kinase has multiple substrates for activation, including tau.

Tau hyperphosphorylation can lead to the formation of NFTs in the AD brain, and pathological tau-induced neurotoxicity therefore plays a key role in driving neurodegenerative dysfunction [28]. Some studies have reported that overactivation of kinases and inactivation of phosphatases can lead to tau hyperphosphorylation [29], such as glycogen synthase kinase-3*β* (GSK-3*β*), Janus kinase (JNK), mitogen-activated protein kinase (MAPK), and Ca^2+^/calmodulin-dependent kinase [30, 31]. In previous studies, TianDiJingWan treatment significantly reduced p-tau aggregation and improved learning and memory in an AD rat model [32], and the A*β*-N-terminus monoclonal antibody (mAb) A8 decreased levels of both A*β* and p-tau in the brains of APP/PS1 mice and alleviated cognitive dysfunction [33]. Therefore, the inhibition of tau hyperphosphorylation can meaningfully improve both neuronal damage and cognitive function.

The HPTQ used in the current study was mainly composed of Radix Rhei Et Rhizome, Chuanxiong Rhizoma, Panax Ginseng C. A. Mey, Acori Tatarinowii Rhizoma, Fructus Alpinae Oxyphyllae, and Radix Polygoni Multiflori Praeparata. Modern pharmacological studies have indicated that ginseng is an effective drug for protecting the nervous system and that it can improve and relieve stress and fatigue and prevent neurodegenerative diseases [34]. Ginsenoside Rg1, an active ingredient of ginseng, was reported to improve cognitive impairment and memory in SAMP8 mice and may regulate PKA/CREB activity via the blood-brain barrier [35]; the effective ingredients of rhubarb are anthraquinone derivatives, including emodin, aloe-emodin, chrysophanol, rhein, physcion, and danthron, and these can improve central nervous system diseases such as traumatic brain injury, brain tumors, and Alzheimer's disease [36]. Furthermore, *β*-asarone can improve cognitive function by suppressing neuronal apoptosis in an AD rat model induced by A*β* [37], and combined with tenuigenin, it can significantly improve the efficacy of memantine in treating moderate-to-severe AD [38]. Tetrahydroxystilbene glucoside (TSG) is the major active component of *Polygonum multiflorum*, which can improve cognitive deficits in AD rats [39]. Ferulic acid, an antioxidant, is the main component of *Ligusticum chuanxiong*, which can protect the brain from A*β* neurotoxicity and neuronal death caused by ROS [40].

Results from our study suggest that the A*β* toxin activates the CaM-CaMKIV pathway, leading to tau hyperphosphorylation, whereas HPTQ and the positive control NMDP attenuated this effect. Moreover, treatment with KN-62 only, as well as cotreatment with HPTQ and KN-62, significantly inhibited tau phosphorylation. However, there were no statistically significant differences between treatment with HPTQ only and cotreatment with HPTQ and KN-62. These results imply that HPTQ protects against hippocampal neuronal damage and tau phosphorylation induced by A*β*_25-35_ and improves learning and memory in AD rats, and suggest that these effects may occur via inhibition of the CaM-CaMKIV pathway.

## 5. Conclusions

For more than 10 years, HPTQ has been used as an in-hospital preparation for the clinical treatment of dementia. However, the mechanisms of its effect are unclear. Our results show that:

1. HPTQ is a multiflavored Chinese medicine composition that contains stilbene glycoside, ferulic acid, ginsenoside Rg1, aloe-emodin, *β*-asarone, emodin, and chrysophanol as its various chemical components
2. HPTQ is protective against hippocampal neuronal damage and reduces hippocampal neuronal loss and acts as a neuroprotective agent in an AD rat model
3. HPTQ reduces tau phosphorylation via inhibition of the CaM-CaMKIV pathway

Overall, our study revealed a potential mechanism of HPTQ in the treatment of AD, thus providing new ideas for the development of therapeutic AD drugs based on eliminating the potentially pathological product tau.

## Figures and Tables

**Table 1:** Gene primer sequences of qRT-PCR.

Primer	Sequence (5′⟶3′)	Length (*bp*)
CaM-F	CGACTTCCCTGAATTCCTGA	20
CaM-R	TCTGCTGCACTGATGTAGCC	20
CaMKK-F	CTTCAAGACCCACACCAGT	19
CaMKK-R	TGTAGAGTAAGGCCCAACC	19
CaMKIV-F	CCACATGGATACCGCTCAGA	20
CaMKIV-R	TGTTGGTGTGACTGCTGCTG	20
Tau-F	TCCACTGAGAACCTGAAGCA	20
Tau-R	TGTCCTTTGAGCCACACTTG	20
ACTIN-F	CCCGCGAGTACAACCTTCTTG	21
ACTIN-R	GTCATCCATGGCGAACTGGTG	21

## Data Availability

The data used to support the findings of this study are available upon request by contact with the corresponding author.
